# Human antimicrobial/host defense peptide LL-37 may prevent the spread of a local infection through multiple mechanisms: an update

**DOI:** 10.1007/s00011-025-02005-8

**Published:** 2025-02-11

**Authors:** Daniel Svensson, Bengt-Olof Nilsson

**Affiliations:** Department of Experimental Medical Science, Lund University, BMC D12, 22184 Lund, Sweden

**Keywords:** Antimicrobial peptides (AMPs), Apoptosis, Cathelicidin/LL-37, Host cell cytotoxicity, Infection, Inflammation

## Abstract

**Background:**

Human cathelicidin LL-37 shows activity towards both gram-positive and gram-negative bacteria, and it is also active against some types of viruses. Besides its antimicrobial effects, the peptide modulates innate immunity through binding and inactivation of bacterial endotoxins and promoting chemotaxis of immune cells.

**Results:**

LL-37 is reported to interact with plasma membrane receptors and mediate import of Ca^2+^. Importantly, LL-37 has both anti- and pro-inflammatory effects. LL-37 is cytotoxic to many different human cell types, particularly infected cells, when administered to the cells at final concentrations of 1–10 µM. In psoriatic lesions very high concentrations (300 µM) of the peptide are detected, and in periodontitis, gingival crevicular fluid contains about 1 µM LL-37, implying high concentrations of the peptide at the site of infection/inflammation which can affect host cell viability locally.

**Conclusions:**

Altogether, LL-37 may inhibit and prevent the infection from spreading by direct anti-bacterial and anti-viral effects, but also via anti- and pro-inflammatory mechanisms, and through killing already infected and weakened host cells at the site of infection/inflammation.

## Introduction

**Fig. 1:**
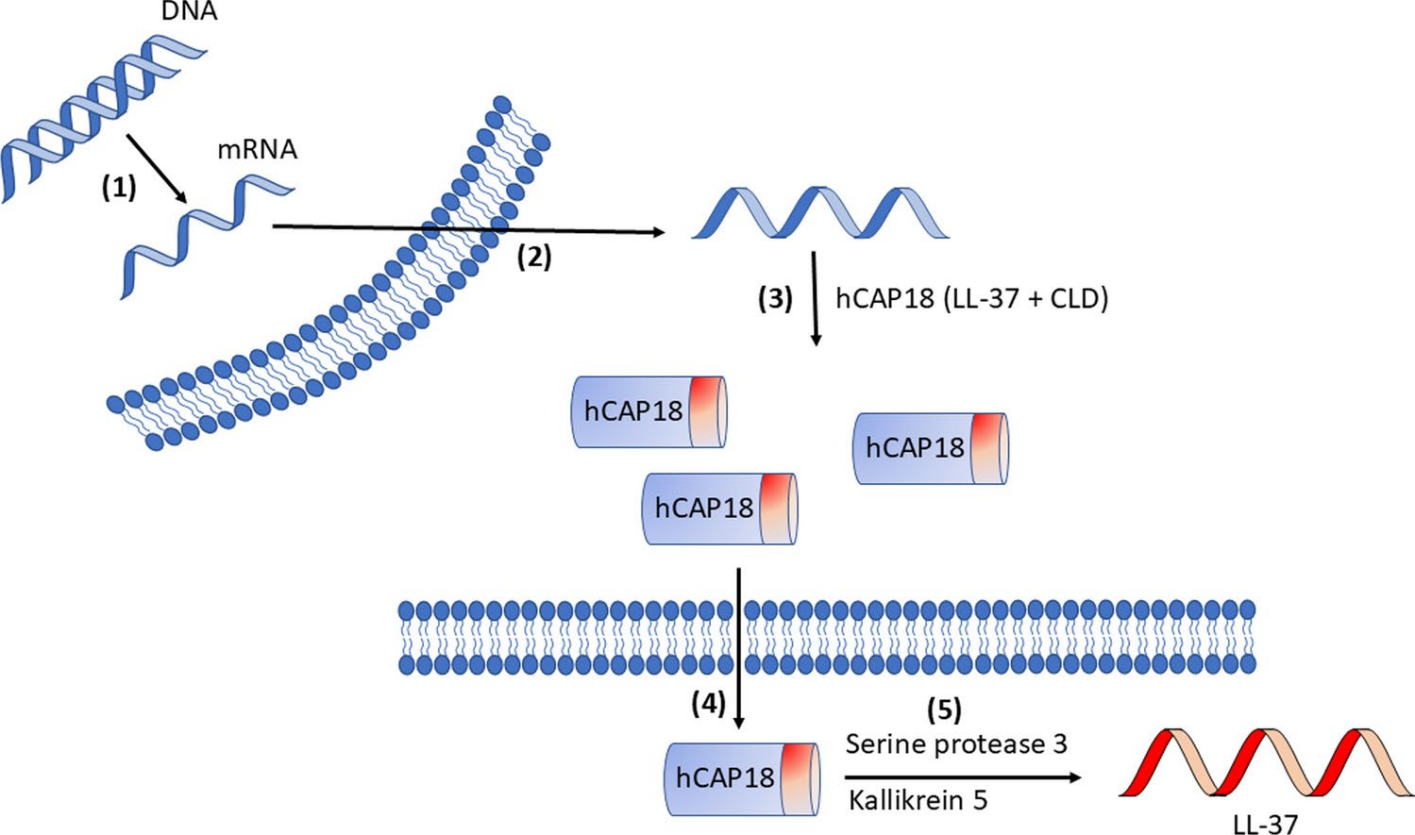
Production and processing of hCAP18/LL-37. White blood cells and epithelial cells secrete hCAP18 which is cleaved extracellularly to LL-37 by serine protease 3 and kallikrein 5. (1) Transcription of the Cathelicidin AntiMicrobial Protein (CAMP) gene, (2) Export of mRNA, (3) Synthesis of hCAP18, (4) Exocytosis of hCAP18, (5) Cleavage of hCAP18 into biologically active LL-37. hCAP18 = human Cathelicidin Antimicrobial Protein 18, CLD = Cathelin-Like Domain

In humans, there are two important families of antimicrobial peptides, cathelicidins and defensins. The human cathelicidin LL-37 is the focus of this review. LL-37 is active against microorganisms and therefore characterized as an antimicrobial peptide, but due to its multiple effects in humans, it is often described as a host defense peptide. The pro-form of LL-37, hCAP18, is secreted by white blood cells and epithelial cells and processed to LL-37 extracellularly through serine protease 3 and kallikrein 5 [1–5]. The formation and processing of hCAP18/LL-37 is depicted in Fig. 1. LL-37 has a rapid turnover, and the systemic levels of LL-37 are low, although elevated levels of LL-37 can be found locally at the infection/inflammation [6–8].

Neutrophils are especially rich in hCAP18, and patients suffering from a congenital severe form of neutropenia (Kostmann syndrome) have extremely low levels/lack LL-37 in blood and saliva [9]. These patients may die from banal infections, if not treated with granulocyte-colony stimulating factor (G-CSF) to elevate their neutrophil contents. Hence, the disease characteristics and symptoms observed in patients with Kostmann syndrome can, at least partly, be caused by lack of sufficient amounts of LL-37, arguing that LL-37 indeed is important in vivo*.*

LL-37 can interact with many cellular structures, molecules and receptors and shows pleiotropic actions [5]. Importantly, the peptide is active against both bacteria and viruses [4, 10]. It has a direct bactericidal effect through permeabilization of the bacterial cell wall, and indirect anti-bacterial effects via binding and inactivation of bacterial endotoxins such as LPS and LTA, and through its ability to dissolve bacterial biofilms and promote formation of neutrophil extracellular traps (NETs). Moreover, LL-37 modulates innate immunity through both pro- and anti-inflammatory mechanisms and by stimulating recruitment of white blood cells [5, 11]. Last, but not least, LL-37 is cytotoxic to many types of human host cells, which may be recognized as a negative side effect, but importantly there are studies showing that LL-37 primarily kills already infected and weakened cells. Here, LL-37-induced cytotoxicity is extensively reviewed and references provided. Thus, it seems that LL-37 can combat microorganisms and simultaneously destroy and remove host cells locally, probably to reduce/eliminate the spread of a local infection. In this review article, we describe and discuss effects of LL-37 on host cells, and we primarily cover and cite articles presenting data from studies using human cells.

## LL-37-induced anti-bacterial effects

LL-37 was originally identified as an anti-bacterial peptide, and the mechanisms by which LL-37 exerts this effect have been studied extensively [10]. The peptide shows toxicity towards both gram-negative and gram-positive bacteria, and it acts via permeabilization of the bacterial cell wall [4, 10]. This effect is completely dependent on its helical structure and observed only at sufficient ionic concentrations. The importance of the direct LL-37-induced bactericidal effect has been debated, since relatively high LL-37 concentrations are needed for bacterial clearance. Notably, the sensitivity to LL-37 varies between different bacterial strains, probably because structure and functional characteristics of the bacterial cell wall vary between bacteria [10]. However, LL-37 also has other anti-bacterial effects, besides causing cell lysis, which most likely are of greater biological importance. This includes its ability to dissolve bacterial biofilms at 100-fold lower concentrations than needed to kill the type of bacteria constituting the bacterial component of the biofilm, as well as its chemotactic properties, involved in the recruitment of immune cells to the site of infection [5, 12].

## LL-37-induced anti-viral and anti-fungal effects

In accordance with LL-37-induced cytotoxicity in host- and bacterial cells equipped with plasma membrane/cell wall, LL-37 is also able to damage enveloped viruses, such as Influenza A, by disintegration of their envelope, allowing for clearance of viral particles by antibodies [13]. Interestingly, vitamin D shows activity against *Mycobacterium tuberculosis* and inhibits HIV virus replication via LL-37-induced autophagy in macrophages [14].

LL-37 can also function as a fungicide through its ability to disrupt fungal membranes; for the main fungal pathogen in humans, *Candida albicans*, LL-37 kills the fungi at concentrations between 0.8 and 8 µM [15, 16]. Besides this effect, it also suppresses growth of various fungi through different mechanisms involving disruption of ER homoeostasis and induction of oxidative stress [17]. Additionally, LL-37 may bind to carbohydrates in the fungal membrane, and by doing so it attenuates fungal adhesion to host cells, which represents the first step in fungal infections [16].

## Anti-inflammatory properties of LL-37

LL-37 shows anti-bacterial and anti-inflammatory capacities also via other mechanisms than direct effects on bacteria and viruses as reviewed previously by Hancock et al. [5]. In Table 1, we summarize LL-37-induced anti- and pro-inflammatory effects and mechanisms of action in various human cell types. The peptide binds and inactivates lipopolysaccharide (LPS) and lipoteichoic acid (LTA) from gram-negative and gram-positive bacteria, respectively. The mechanism behind this effect is regarded to be the direct binding of cationic LL-37 to negatively charged LPS and LTA and thereby inactivation of the endotoxins [18, 19]. LL-37 inhibits TNF-α production induced by LPS from many types of bacteria, such as *S. typhinarium*, *B. cepacia* and *E. coli*, implying that LL-37-induced inactivation of LPS is independent of the bacterial source of LPS [19]. LL-37 inhibits the pro-inflammatory effect of LPS in macrophages and epithelial cells, but also in other human cell types such as periodontal ligament cells, where it has been shown to prevent LPS-induced MCP-1 production [20]. Besides its binding and inactivation of endotoxins and thereby inhibition of endotoxin-mediated inflammation, LL-37 is reported to trigger production of the anti-inflammatory cytokines IL-1RA and IL-10 [21, 22]. Overall, these reports suggest that LL-37 acts anti-inflammatory through multiple mechanisms.

**Table 1:** LL-37-induced pro- and anti-inflammatory mechanisms in different human cell types. Notably, in some types of cells LL-37 shows both pro- and anti-inflammatory effects

Cell type	Pro-inflammatory	Anti-inflammatory	Mechanism	Reference
Erythrocytes, Macrophages		Yes	Binding and inhibition of LPS	[18]
Macrophages, Epithelial cells	Yes	Yes	Pro: Up-regulation of chemokines Anti: Inhibition of bacterial endotoxins	[19]
PBMCs	Yes		Potentiates IL-1β-induced cytokine production	[26]
Monocytes		Yes	Enhances anti-inflammatory IL-10	[22]
Keratinocytes		Yes	Inhibits cytosolic DNA-induced inflammasome	[36]
Bronchial epithelial cells	Yes		Potentiation of poly I:C-induced TLR3 signaling	[29, 30]
Macrophages	Yes		Stimulates activation of the inflammasome	[32, 33]
Keratinocytes	Yes	Yes	Pro: Potentiates poly I:C-induced IL-8 Anti: Inhibits poly I:C-stimulated CCL5 and CXCL10	[31]
Bronchial epithelial cells	Yes		Recruits eosinophils	[24]
Macrophages		Yes	Triggers anti-inflammatory cytokine IL-1RA	[21]
Vascular smooth muscle cells	Yes		Potentiates poly I:C-induced inflammation via up-regulation of TLR3	[28]
Colonic epithelial cells	Yes		Potentiation of LPS-induced IL-8	[25]
Gingival fibroblasts	Yes	Yes	Pro: Stimulation of IL-8 and CXCL1 expression Anti: Inhibition of LPS-induced IL-6 and IL-8	[23]

## Pro-inflammatory properties of LL-37

In macrophages and gingival fibroblasts, LL-37 has been shown to up-regulate transcripts for chemokines and chemokine receptors [19, 23]. LL-37 triggers chemokine production by airway epithelial cells and colonic epithelial cells, and promotes recruitment of eosinophils to bronchial epithelial cells, implying a significant role for LL-37 in chemotaxis [24, 25]. Yu et al. [26] show that LL-37 potentiates pro-inflammatory IL-1β-induced cytokine and chemokine production (for example the chemokine MCP-1) by peripheral blood mononuclear cells (PBMCs). Furthermore, LL-37 stimulates pro-inflammatory activity and promotes migration of tissue mast cells [27]. Altogether, these data suggest that LL-37 triggers recruitment of immune cells to the inflammatory process.

In vascular smooth muscle cells, LL-37 potentiates the pro-inflammatory effect of synthetic double-stranded RNA (poly I:C) via up-regulation of TLR3, suggesting that LL-37 promotes virus-induced inflammation [28]. Also, in bronchial epithelial BEAS-2B cells, LL-37 enhances poly I:C-induced inflammation [29, 30]. In keratinocytes, LL-37 is reported to amplify poly I:C-induced IL-8 chemokine expression, whereas it antagonizes poly I:C-stimulated thymic stromal lymphopoietin cytokine and CCL5 and CXCL10 chemokine expressions [31]. In summary, LL-37 seems to potentiate poly I:C-induced inflammation.

LL-37 stimulates the NLRP3-mediated inflammasome in macrophages and induces skin inflammation in mice via activation of the inflammasome [32, 33]. In mice, LL-37 is reported to worsen myocardial injury, and this effect appears to involve activation of the NLRP3 inflammasome [34]. In keratinocytes, LL-37 potentiates UVB radiation-induced inflammasome mediated production of IL-1β [35]. Hence, several studies demonstrate that LL-37 triggers the NLRP3 inflammasome and inflammation. On the other hand, LL-37 has been shown to antagonize cytosolic DNA-induced inflammasome activation through the DNA sensor AIM2, suggesting that LL-37 may interact with the inflammasome and act anti-inflammatory in sterile inflammation caused by DNA [36].

## LL-37-induced host cell toxicity

It is well-documented that µM concentrations of exogenous LL-37 cause morphological alterations such as cell shrinkage, increase lactate dehydrogenase (LDH) release and reduce cell viability assessed by for example the MTT assay in various types of human host cells. These effects, which are signs of reduced cell viability and apoptosis in response to LL-37, can be seen in the concentration range 1–10 µM. LL-37-induced cytotoxic and pro-apoptotic effects are observed in cells treated with synthetic LL-37 from different manufacturers, and in concentrations which are relevant for the in vivo situation [6–8]. In Table 2, we present studies reporting reduced cell viability in response to LL-37 in different human cell types, and examples of techniques used to demonstrate LL-37-induced cytotoxicity and apoptosis. Interestingly, Barlow et al. [37] show that LL-37 acts in synergy with *P. aeruginosa* to promote apoptosis in human bronchial epithelial cells, and hence LL-37 seems to trigger apoptosis preferably in infected cells, but LL-37-induced pro-apoptotic effects are also observed in non-infected airway epithelial cells [38]. Thus, LL-37 appears to favor already weak and injured host cells as target cells for LL-37-induced cytotoxicity. On the same theme, Björstad et al. [39] demonstrate that LL-37 primarily permeabilizes the plasma membrane of already apoptotic neutrophils, whereas it has weak or no effect in healthy cells. As pointed out before, LL-37 is a cationic peptide binding to negative moieties of plasma membranes, suggesting that infected and injured cells mobilize and expose negative charges to the outer surface of their plasma membrane. The degree of cytotoxicity caused by LL-37 varies between cell types. For example, LL-37 has a more powerful cytotoxic effect in periodontal ligament cells and osteoblasts compared to keratinocytes and monocytes [40, 41]. In nasal epithelial cells, LL-37 induces cell death via non-apoptotic processes which probably involve cell necrosis, further demonstrating that LL-37 is cytotoxic via different cell specific mechanisms [42]. Thus, LL-37 may cause cell death through different pathways depending on cell type, and likely also concentration and time of exposure to LL-37 are important factors.

**Table 2:** LL-37-induced human host cell cytotoxicity and apoptosis. Here, we present cell types and assays and techniques used to demonstrate LL-37-induced cytotoxicity and pro-apoptotic effects of LL-37. NA = not available

Cell type	Cell viability assay	Cell number/counting assay	Apoptosis assay	Ref
Vascular smooth muscle cells	FITC-annexin V microscopy LDH release	NA	Caspase-3 activity DNA fragmentation ELISA Flow cytometry	[58]
Airway epithelial cells	NA	NA	Cleaved caspase-3 and 9 Cytochrome C release TUNEL staining	[37]
Periodontal ligament fibroblasts	Microscopy of live cells	DNA synthesis Counting in Bürker chamber	Active caspase-3 ELISA	[59]
Airway epithelial cells	MTT assay	NA	Cytochrome C release TUNEL staining Active caspase-3 and 7 PARP cleavage	[52]
Regulatory T cells	NA	NA	DNA fragmentation Chromatin condensation Apoptotic body formation	[54]
MG63 osteoblasts	LDH release MTT assay Microscopy of live cells	NA	NA	[40]
HaCaT keratinocytes	LDH release MTT assay Microscopy of live cells	NA	NA	[40]
MG63 osteoblasts	Microscopy trypan blue	Counting in Bürker chamber	Active caspase-3 ELISA Flow cytometry Annexin V	[49]
Airway epithelial cells	LDH release	NA	TUNEL staining Active caspase-3 Western blot	[60]

In summary, LL-37 reduces viability and causes apoptosis in most human cell types, although its impact differs between cell types. To our knowledge, LL-37-induced anti-apoptotic effects have only been observed in keratinocytes, dermal fibroblasts, and neutrophils, suggesting that this effect is coupled to specific properties of these cell types and/or experimental conditions [38, 43–45]. In neutrophils, LL-37 reduces cell turnover by causing secondary necrosis demonstrated by flow cytometry of cells stained with propidium iodide (PI) and annexin V. Here, LL-37 rapidly converts the neutrophils from PI-negative, annexin V-positive cells to PI-positive cells, i.e., transform them into necrotic cells, and this effect does not seem to be pro-inflammatory to macrophages [46, 47].

## LL-37 induces caspase-independent apoptosis

**Fig. 2:**
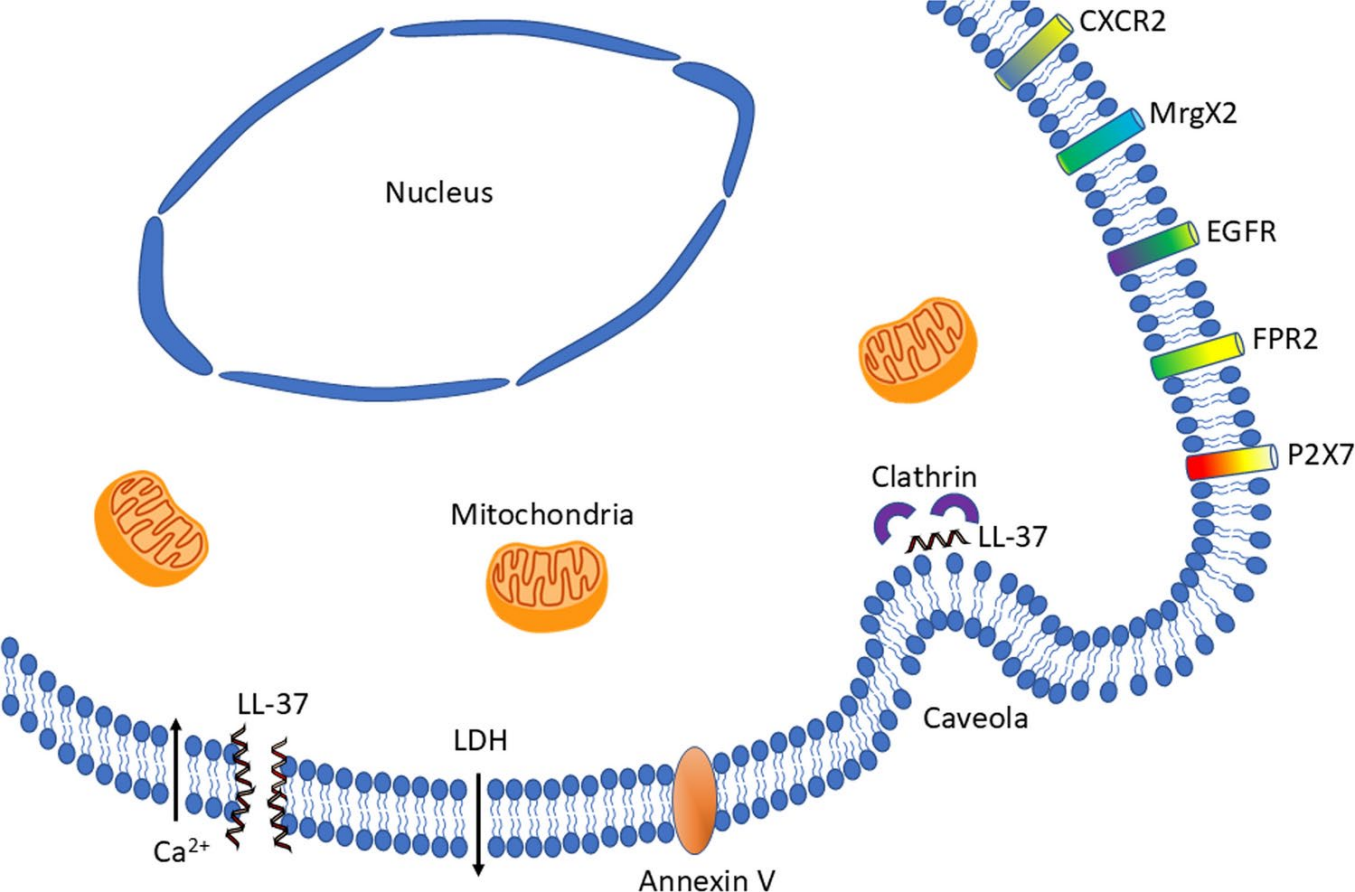
Interactions of exogenous LL-37 with the plasma membrane of human host cells. LL-37 causes inflow of Ca^2+^, release of LDH, pore formation and flip of Annexin V indicative of early apoptosis. LL-37 is supposed to be imported via both clathrin- and caveolae-mediated endocytosis. Moreover, LL-37 is believed to exert its effects via G protein-coupled receptors, receptor tyrosine kinases and purinergic receptors as shown in the upper right part of the figure

In Fig. 2, we depict how exogenous LL-37 may interact with the plasma membrane in human host cells. LL-37 causes pore formation, allows for inflow of Ca^2+^, promotes LDH release and enhances the proportion of annexin V positive cells [40, 48–50]. Moreover, LL-37 is believed to be imported via both clathrin- and caveolae-mediated endocytosis as described in Fig. 2, and hereby it may bind and interact with intracellular binding partners [51]. LL-37-induced flip of annexin V indicates early apoptosis, and moreover TUNEL positive cells are seen in response to LL-37, showing that LL-37 causes DNA fragmentation, and late stage apoptosis. LL-37-induced apoptosis is not associated with caspase-3 activation or PARP cleavage in Jurkat cells, airway epithelial cells and osteoblast-like cells (MG63 cells), suggesting that LL-37 causes caspase-independent apoptosis in these cell types [37, 50, 52]. In oral squamous carcinoma cells, a C-terminal domain of hCAP18 (hCAP18_109-135_) has been reported to trigger caspase-independent apoptosis [53]. Although LL-37 induces apoptosis that is not associated with caspase activity in healthy epithelial cells, it may cause caspase-3 activation in epithelial cells infected with bacteria [37]. In regulatory T cells, LL-37 causes both caspase-dependent and caspase-independent apoptosis, suggesting that both processes may occur in this cell type [54]. Mader et al*.* [55] show that LL-37-induced apoptosis is dependent on apoptosis-inducing factor (AIF) released from mitochondria in Jurkat cells, implying that LL-37 directly and/or indirectly interacts with mitochondria and thereby triggers AIF-dependent apoptosis. In colon cancer cells, LL-37 causes caspase-independent apoptosis and translocation of AIF from cytosol to nucleus, and in mitochondria isolated from MG63 cells, LL-37 releases AIF, providing further evidence that AIF from mitochondria is responsible for LL-37-induced apoptosis [56, 57]. Although, results of many studies imply that caspase-independent apoptosis caused by mitochondrial AIF is responsible for LL-37-induced cell death, further studies are needed to clarify the importance and involvement of caspase-dependent apoptosis, caspase-independent apoptosis, necrosis and other alternative mechanisms in cell death of human host cells caused by LL-37. In Table 2, we include additional studies, not discussed in the text, reporting cell death in response to treatment with exogenous LL-37 [58–60].

## LL-37 signaling through plasma membrane receptors

Besides LL-37-induced permeabilization of the plasma membrane allowing for influx of Ca^2+^ from the extracellular space as discussed before, LL-37 is also believed to interact with the host cell plasma membrane via G protein-coupled receptors (GPCRs), receptor tyrosine kinases and purinergic receptors as illustrated in Fig. 2. Although LL-37 is suggested to exert its action via plasma membrane receptors, it is unclear if this represents a direct interaction with the extracellular ligand-binding domain of the receptor, or if the peptide acts through indirect mechanisms, e.g., by interaction with the cytoplasmic portion of the receptor and/or through its pore forming capacity [61]. Treatment with pertussis toxin, causing disruption of GPCR signaling, has been reported to inhibit LL-37-induced apoptosis in colon cancer cells, implying that this effect is mediated by a GPCR [56]. Over the years, a considerable number of different GPCRs are suggested to mediate LL-37 downstream effects. In mast cells, the GPCR, Mas-related gene X2 (MrgX2), is reported to mediate LL-37-evoked mast cell degranulation [62]. CXCR2 is a GPCR that acts as a receptor for the chemokine interleukin 8, and LL-37 is reported to act as a functional ligand for this receptor in human neutrophils [63]. The formyl peptide receptor 2 (FPR2) is a GPCR expressed by many different human cell types including white blood cells. The arachidonic acid metabolite lipoxin A4 as well as bacterial/viral derived peptides are proposed to act as FPR2 ligands which upon binding trigger, for example chemotaxis. LL-37 has been reported to activate MAPK via FPR2 in cancer cells, and FPR2 is also suggested to play a role in LL-37-stimulated chemoattraction of white blood cells [64–66]. Recently, Lou et al. [67] show that LL-37 upregulates FPR2, activates opening of mitochondrial permeability transition pores, and stimulates formation of NETs in neutrophils. Interestingly, LL-37 increases synthesis of FPR2 in human mesenchymal stem cells (hMSC), allowing for FPR2-mediated chemokine/cytokine production and accumulation of hMSCs in atherosclerotic plaques [68]. LL-37 promotes phagocytosis by human macrophages, and this effect is also suggested to be mediated through FPR2 [69]. Moreover, LL-37 has been reported to promote wound healing and epithelial cell proliferation of airway epithelium through activation of EGFR, belonging to tyrosine kinase membrane receptor family [70].

The ionotropic P2X7 receptor belongs to the family of purinergic receptors, and it is activated by ATP. However, P2X7 receptors are also suggested to mediate LL-37 signaling. In this paragraph, we present examples of LL-37-induced responses in human cells reported to involve the P2X7 receptor. In human macrophages, LL-37-induced stimulation of thromboxane A_2_ and leukotriene B_4_ formation, and LL-37-stimulated ERK phosphorylation, are reported to involve P2X7 [71]. Furthermore, LL-37 is suggested to be internalized by macrophages through clathrin- and caveolae-mediated endocytosis via activation of P2X7 receptors [33, 51]. In human monocytes, Rekha et al. [72] show that the P2X7 receptor antagonist KN62 inhibits LL-37-stimulated autophagy, suggesting that P2X7 may be involved in this effect. A recent study reports that LL-37-induced autophagy is dependent on post-translational modification of LL-37, and interestingly this effect is observed at low concentrations of LL-37 (2 µg/ml = 0.44 µM), where no obvious cytotoxic effects of LL-37 are observed [40, 73]. In human gingival fibroblasts, P2X7 receptors seem to be involved in LL-37-induced stimulation of interleukin-8 expression [74]. Stiffening of endothelial cells induced by LL-37 may represent an anti-inflammatory mechanism, and this effect is suggested to be blocked by P2X7 receptor antagonists, arguing that LL-37-evoked endothelial cell stiffening is dependent on P2X7 receptor signaling [75]. In human neutrophils, LL-37 has been reported to exert an anti-apoptotic effect as discussed previously, and this effect is suggested to be mediated via FPR2 and P2X7 receptors [45]. Finally, P2X7 has been implicated in LL-37-induced IL-1β production by monocytes [76]. Hence, the P2X7 receptor is reported to mediate various LL-37-induced effects in many different cell types.

## Conclusions

LL-37 combats bacteria and viruses, both directly through permeabilization of their cell wall/envelope, and indirectly via binding and inactivation of bacterial endotoxins and stimulation of inflammation. In µM concentrations, LL-37 permeabilizes cell membranes and is cytotoxic to human host cells, especially infected and injured cells, and it also induces apoptosis. Therefore, LL-37 may kill tissue cells, at the site of infection/inflammation, where the local concentration of the peptide is high. The main purpose of this review is to summarize these diverse effects of LL-37 and highlight that they may act in synergy to combat pathogens. More information about systemic and local levels of LL-37 in healthy individuals and in patients with inflammatory/infectious diseases represents an important topic for future studies. Another important matter is to better clarify LL-37-evoked signaling pathways and their complex down-stream effects and importance in inflammation. In local infections, LL-37 may thus eliminate infected host cells and thereby cause local tissue destruction and loss of functionality. However, on the systemic level, these mechanisms can help the patient avoid a spread of the infection and life-threatening sepsis. Importantly, there is no direct evidence that LL-37-induced cytotoxicity is beneficial. Therefore, it is important to examine the multiple actions of LL-37 in complex experimental set-ups, such as co-culture systems with many cell types, and of course in vivo, to get a more complete picture of its integrated and systemic role.

## Data Availability

No datasets were generated or analysed during the current study.
